# Battling the Bots and Defending Against Fraudulent Responses in an International Community-Engaged Web-Based Survey With People Living With Long COVID: Methodological Study

**DOI:** 10.2196/88838

**Published:** 2026-07-23

**Authors:** Kiera McDuff, Tai-Te Su, Darren A Brown, Jessica M Martin, Soo Chan Carusone, Sarah O'Connell, Imelda O'Donovan, Natalie St. Clair-Sullivan, Liam Townsend, Susie Goulding, Mary Kelly, Lisa McCorkell, Hannah Wei, Margaret O'Hara, Leticia Soares, Lisa Avery, Ciaran Bannan, Colm Bergin, Richard Harding, Julia Nathanson, Patricia Solomon, Angela M Cheung, Jaimie Vera, Kelly K O'Brien

**Affiliations:** 1 Department of Physical Therapy, Temerty Faculty of Medicine, University of Toronto, 160-500 University Avenue, Toronto, ON, M5G 1V7, Canada; 2 School and Graduate Institute of Physical Therapy, College of Medicine, National Taiwan University, Taipei, Taiwan; 3 Chelsea and Westminster Hospital NHS Foundation Trust, London, United Kingdom; 4 Long COVID Physio, London, United Kingdom; 5 McMaster Collaborative for Health and Aging, McMaster University, Hamilton, ON, Canada; 6 Long COVID Advocacy Ireland, Dublin, Ireland; 7 Cicely Saunders Institute, Florence Nightingale Faculty of Nursing Midwifery and Palliative Care, King's College London, London, United Kingdom; 8 Department of Infectious Diseases, St. James's Hospital, Dublin, Ireland; 9 Department of Clinical Medicine, Trinity College Dublin, St. James's Hospital, Dublin, Ireland; 10 COVID Long Haulers Support Group, Cambridge, ON, Canada; 11 Patient-Led Research Collaborative, Oakland, CA, United States; 12 Patient-Led Research Collaborative, Ottawa, ON, Canada; 13 Long Covid Support UK, Birmingham, United Kingdom; 14 Department of Biostatistics, Princess Margaret Cancer Centre, University Health Network, Toronto, ON, Canada; 15 Dalla Lana School of Public Health, University of Toronto, Toronto, ON, Canada; 16 School of Rehabilitation Science, Faculty of Health Sciences, McMaster University, Hamilton, ON, Canada; 17 Institute of Health Policy, Management and Evaluation (IHPME), Dalla Lana School of Public Health, University of Toronto, Toronto, ON, Canada; 18 Department of Medicine, Temerty Faculty of Medicine, University of Toronto, Toronto, ON, Canada; 19 Department of Medicine, University Health Network, Toronto, ON, Canada; 20 Department of Global Health and Infection, Brighton and Sussex Medical School, University of Sussex, Brighton, United Kingdom; 21 Brighton and Sussex University Hospitals NHS Trust, Royal Sussex Hospital, Brighton, United Kingdom; 22 Rehabilitation Sciences Institute, University of Toronto, Toronto, ON, Canada

**Keywords:** survey, questionnaire, online research, survey fraud, social media, artificial intelligence, bots

## Abstract

**Background:**

Web-based surveys involving self-reported questionnaires are vulnerable to fraudulent responses. Advancements in artificial intelligence and bots have introduced additional challenges to preventing and identifying fraudulent responses to online questionnaires.

**Objective:**

This study aimed to describe our experiences with fraudulent responses, strategies for preventing and identifying fraudulent responses, lessons learned when conducting a web-based survey with adults living with Long COVID, and recommendations for web-based survey research.

**Methods:**

The Long COVID and Episodic Disability Study is an international community-engaged study among adults living with Long COVID in Canada, Ireland, the United Kingdom, and the United States. We conducted a longitudinal web-based survey, with online administration of a self-reported questionnaire at 2 timepoints (Time 1 and Time 2), 1 week apart. We recruited through Long COVID community groups using social media, emails, and word of mouth. The survey was disrupted by fraudulent responses, including bots. To defend data integrity, we implemented the following strategies: (1) pausing our initial launch (Wave 1), (2) developing and implementing screening criteria to identify fraudulent responses, and (3) relaunching the web-based survey (Wave 2) with revised recruitment strategies and questionnaire design to prevent and identify fraudulent responses.

**Results:**

We received 4663 responses for Time 1 and 1281 responses for Time 2, of which we retained 798 of 4663 (17%) responses and 629 of 1281 (49%) responses. Strategies for preventing fraudulent responses included enabling survey protection features in survey software, shutting down compromised survey links, avoiding recruitment via public social media groups, and removing mention of a financial incentive from recruitment materials. Strategies for identifying fraudulent responses included monitoring response completion times, start and end time stamps, geolocation, and screening for suspicious email address characteristics and duplicates.

**Conclusions:**

Our lessons learned fell into the following three areas: (1) survey-design and implementation to prevent and identify fraudulent and bot-generated responses, (2) recruitment strategies to mitigate the risk of disruption by bots, and (3) responding to disruptions caused by fraudulent and bot responses. We recommend the following tactics to prevent and mitigate the risks of fraudulent and bot responses when administering online web-based questionnaires: (1) review current literature and connect with researchers and Research Ethics Boards about strategies before launching, (2) invest in survey software with rigorous information security technology, (3) use bot-detection features available in survey software before launching, (4) design questionnaire items to identify bots and fraudulent actors, (5) tailor criteria for identifying fraudulent and bot responses to the characteristics of the target population, (6) avoid recruitment in public social media groups, (7) engage community leaders in tailored and targeted recruitment, (8) avoid advertising incentives, (9) shut down compromised links rapidly, (10) communicate with the Research Ethics Board about disruptions, and (11) combine automated and manual methods to identify potentially fraudulent responses on time.

## Introduction

Web-based surveys, defined as surveys administered via online platforms (such as Qualtrics [Qualtrics, LLC], SurveyMonkey [SurveyMonkey Inc], or REDCap [Vanderbilt University Medical Center]) [1-4], have become an increasingly popular and important study design [5-7]. They may involve cross-sectional, observational cohort, or longitudinal study designs. Web-based surveys often involve recruitment through online platforms, including email or social media, and administration of online self-reported questionnaires for data collection [7,8]. With online recruitment methods, participants may not directly communicate with researchers and may remain anonymous [6,9-11].

Although the rising popularity of web-based surveys is attributable to the perceived benefits, there are also disadvantages to web-based surveys. Perceived benefits include that online recruitment and data collection may be more efficient than in-person, telephone, or postal mail-based methods and have the ability to reach diverse populations, including people living with disabilities [5,8,10-12]. Online questionnaires are also relatively low-cost to administer [5,10,11]. However, a disadvantage of web-based surveys is their vulnerability to fraudulent responses, especially if recruitment is conducted on social media [5,13-15]. Recruitment on social media is vulnerable to fraud as it involves a limitless pool of (mostly anonymous) potential participants and may involve limited interaction with researchers [16]. Fraud may significantly impair data integrity and necessitate extensive and time-consuming data cleaning to protect the integrity of the collected data [5,13,15,17-19]. Researchers must consider the limitations of web-based surveys alongside the strengths.

Different types of fraud can impact web-based survey studies. For example, (1) unique participant fraud, defined as individuals who access a survey multiple times intentionally (eg, to obtain compensation) or unintentionally [20]; and (2) alias fraud, defined as a single individual using sophisticated techniques to conceal their identity and submit multiple responses to take advantage of participant incentives [20]. Bots are an example of technology that can be applied to commit alias fraud. Bots are a form of automation scripts designed to execute tasks that require limited human intervention [21]. Programmers customize automation scripts to perform automated tasks, such as completing surveys that offer incentives to participants or infecting computers with malware [5,22].

Bots pose unique threats to web-based survey research [23]. They can rapidly and automatically complete surveys, creating a large volume of invalid data. Bots have disrupted web-based surveys in health services research across a variety of topics, including e-cigarette usage [8] and psychology [22], as well as in a range of populations, including Two-Spirit, lesbian, gay, bisexual, transgender, and queer and questioning communities and people living with HIV [24]. These disruptions to health services research can impair data integrity, generate research that misrepresents the intended research population, and lead to interventions and treatments that are guided by skewed results [23,25]. Furthermore, these disruptions impose time and financial costs on researchers in the form of additional hours cleaning data, rewriting surveys, and rerecruitment and may foster mistrust in research among research populations [6,9,23,25-27].

Generative artificial intelligence (AI) has made it more challenging to identify bot-generated responses to online questionnaires [15,28-30]. Research is emerging on the potential benefits of AI for health services research, such as enhanced accuracy of data analysis and optimization of resources through efficiency [31]. However, generative AI has disrupted previously effective means of identifying bot-generated responses to online questionnaires. Before recent advancements in generative AI, bots tended to follow a consistent and identifiable pattern when responding to web-based surveys (such as gibberish or irrelevant answers to open-ended questions, similarly formatted email addresses, and relatively short survey completion times), which could be used to differentiate their responses to web-based surveys from those of humans [15,29]. AI-guided bots now can generate human-like responses to surveys, increasing the challenge of differentiating valid from invalid responses [15,28-30]. As researchers have begun to experience disruptions to web-based surveys by AI-generated bots, methods are evolving to address and prevent this threat to data integrity [15,20,23,26,32]. However, as bot technology and programming continue to evolve, there is a need for researchers to continue implementing adjustments to prevention and mitigation strategies [23,27-30,33].

Bot and AI technology is rapidly evolving, posing complex challenges to maintaining data integrity for web-based surveys [5,15,17,18]. Authors of a 2025 scoping review [34] on identifying and counteracting fraudulent responses generated through online recruitment for health research concluded that existing research on strategies to mitigate fraud in online health research is insufficient to provide evidence-based guidance on the effectiveness of those strategies. While in-person, telephone, and postal mail-based survey methods may pose less risk of fraudulent responses, web-based surveys are important for reaching diverse and vulnerable populations, including people living with disabilities [5,8,10-12]. As identified by Pozzar et al [17], “Development and testing of novel strategies to prevent and detect fraud is a research priority.”

Our aim is to describe our experiences conducting an international community-engaged web-based survey with adults living with Long COVID in Canada, Ireland, the United Kingdom, and the United States. Specifically, we describe (1) our experiences with fraudulent and bot responses, (2) our iterative strategies to prevent and to detect fraudulent and bot responses, and (3) our lessons learned and recommendations to prevent and mitigate fraudulent and bot responses in web-based survey research.

## Methods

### Study Design

We conducted a community-engaged longitudinal measurement study using a web-based survey study design involving electronic administration of 2 questionnaires, approximately 1 week apart, with adults living with Long COVID in Canada, Ireland, the United Kingdom, and the United States [35].

### Context

The aim of the Long COVID and Episodic Disability Study is to advance the conceptualization and measurement of episodic disability among adults living with Long COVID to inform clinical practice, research, and policy [35-37]. This paper will focus on the second phase of this study, for which the aim was to describe health challenges experienced among adults living with Long COVID and assess the measurement properties of a self-reported disability questionnaire, the Episodic Disability Questionnaire (EDQ) [35]. This phase of the study involved online recruitment of adults living with Long COVID in Canada, Ireland, the United Kingdom, and the United States and electronic administration (via Qualtrics) of 2 online questionnaires (Time 1 and Time 2) [1,35]. Respondents were asked to indicate their email address in the Time 1 questionnaire. We sent a Qualtrics link to a second online questionnaire (Time 2) to the email addresses provided at Time 1, 1 week after completing Time 1 [1]. The questionnaires were estimated to require 30‐40 minutes and 10‐15 minutes to complete for Times 1 and 2, respectively.

### Participants

We included adults (aged ≥18 years) living in Canada, Ireland, the United Kingdom, and the United States, who self-identified as living with Long COVID, with access to a computer, tablet, or smartphone and internet [38]. We included individuals with suspected or confirmed acute COVID-19 irrespective of whether they were hospitalized or had a positive or negative SARS-CoV-2 test (polymerase chain reaction, antigen, or antibody) [38].

### Initial Recruitment Strategy

On January 11, 2024, we launched recruitment of participants through online Long COVID community groups (COVID Long Haulers Support Group Canada [39], Long COVID Advocacy Ireland [40], Long COVID Physio [41], Long Covid Support UK [42], and Patient-Led Research Collaborative [43]). Recruitment materials included a poster with a link or QR code to the Time 1 questionnaire, with contact information for the relevant research coordinator, social media posts, and an email to participants from an earlier phase of the study who agreed to be contacted about future research (Multimedia Appendix 1). Information provided in the recruitment materials included a brief description of the study, the inclusion criteria, and indicated that a token of appreciation (a CAD $40 [CAD $1=US $0.72 as of June 15, 2026]) or equivalent currency, an Amazon electronic gift card) would be offered to participants upon completion of the Time 2 questionnaire (Multimedia Appendix 1). Leaders of community groups circulated the study recruitment materials electronically within their networks on social media (including Facebook [Meta Platforms, Inc], Instagram [Meta Platforms, Inc], and X [X Corp]), via email, and word of mouth [44,45]. The social media pages of these groups, some of which were public (ie, no group membership required to view posts), have up to 25,000 followers. Research coordinators emailed the recruitment poster to participants from an earlier phase of the study who provided their contact information and indicated they were interested in participating in future phases of the study [36,37].

### Initial Data Collection Procedures

#### Time 1

We administered an online questionnaire that included (1) the EDQ [46]; (2) Long COVID Episodic Disability Questionnaire Supplement; (3) four criterion measures, including the World Health Organization Disability Assessment Schedule 2.0 [47], Work and Social Adjustment Scale [48], EuroQol 5-Dimensions 5-Levels [49], and modified COVID-19 Yorkshire Rehabilitation Scale [50]; (4) a sensibility questionnaire; and (5) a demographic questionnaire. If an individual participated in Time 1 (and completed ≥50% of the questionnaire), the research coordinator (JMM) emailed a link for the Time 2 questionnaire, 1 week later (Multimedia Appendix 2). We downloaded responses from Qualtrics weekly [1].

#### Time 2

We administered an online questionnaire comprised of the EDQ and Long COVID Episodic Disability Questionnaire Supplement only. At the end of the questionnaire, participants were asked if they wanted to receive a token of appreciation. If they responded “yes,” they were asked to provide their name and email address to receive the token of appreciation (by email).

#### Prelaunch Strategies for Prevention and Detection of Fraudulent Responses

We recognized the potential risk of receiving fraudulent (including bot-generated) responses to our online questionnaire and implemented the following prevention and detection strategies before launching the survey.

#### Fraudulent Response and Bot Prevention

We enabled and used Qualtrics survey protection settings [1], such as preventing multiple responses (a setting that prevents multiple submissions by placing a cookie in the browser of the participant), and reCAPTCHA scores (a question placed before the questionnaire asking the respondent to confirm they are a human).

#### Fraudulent Response and Bot Detection

We monitored the rate and quality of incoming responses. Research coordinators (KM and JMM) manually assessed the quality of responses, one by one, for indicators of potential fraudulent responses (herein referred to as Human Review).

#### Initial Launch of Recruitment: Wave 1

**Figure 1.**
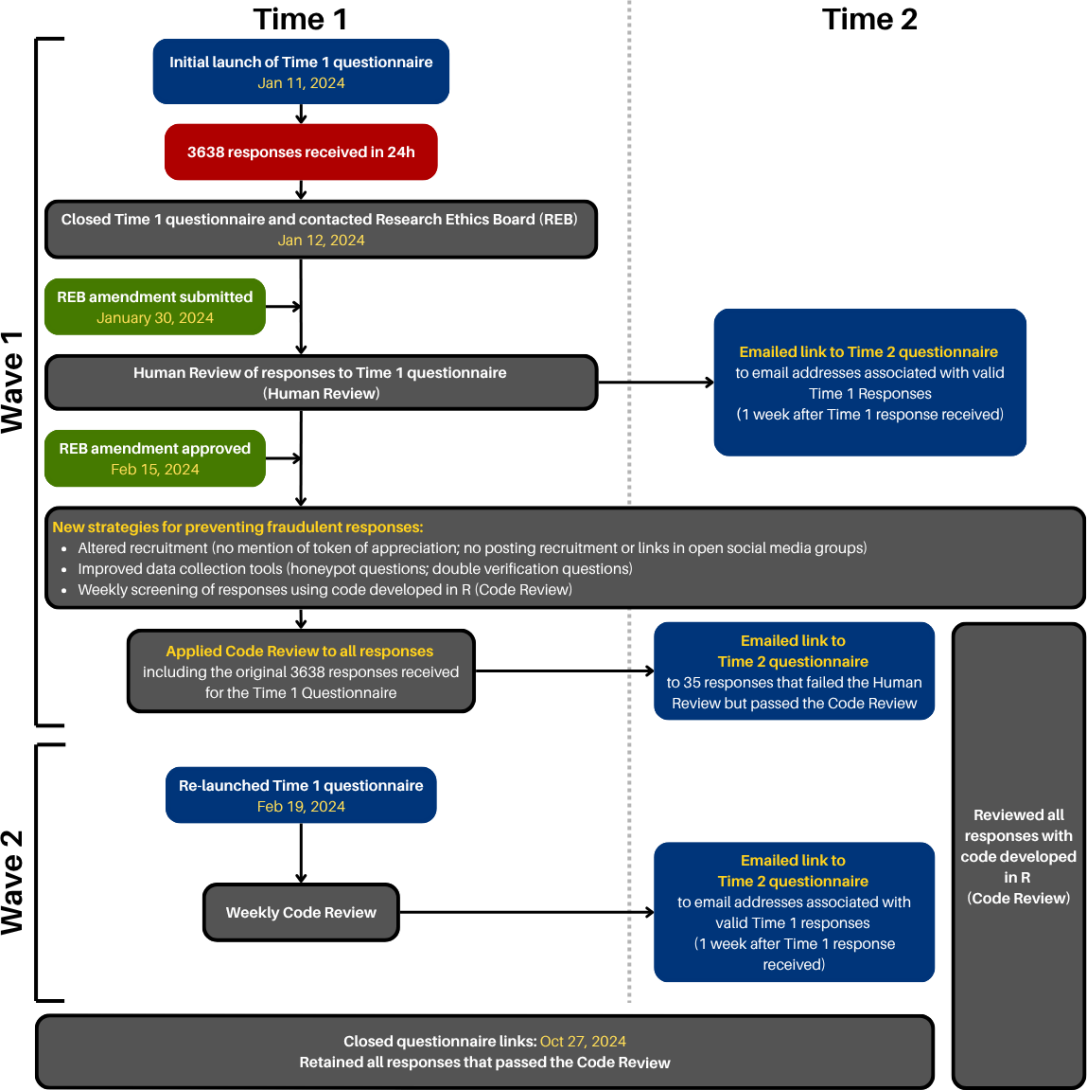
Overview of the timeline of events for survey implementation.

Refer to Figure 1 for an overview of the timeline of events for survey implementation. We launched recruitment on January 11, 2024 (Wave 1). Despite our initial bot prevention and detection strategies, we received a rapid influx of fraudulent responses within the first 24 hours of launch. We closed the link to the Wave 1 (Time 1) questionnaire on January 12, 2024, while allowing responses that were in progress to be completed after we closed the link.

We contacted the Research Ethics Board (REB) at the University of Toronto and sought strategies to protect the integrity of the data and study. We contacted Qualtrics to explore options within the software to detect bot-generated (or other fraudulent) responses [1]. We also conducted a revised literature review on recent strategies for the prevention and detection of fraudulent responses in web-based surveys.

#### Review of Responses

We refined our Human Review of the data to identify suspicious patterns and characteristics that were most likely indicative of fraudulent responses and/or bots, based on available literature and guidance [18-20,22,23,26,28,29,51]. This was time-sensitive, given that the Wave 1, Time 2 questionnaire was to be administered and completed 1 week later. The Human Review involved time-intensive and careful examination of participants’ reported names, email addresses, dates of survey completion, and survey completion duration, with particular attention to identifying repeating patterns and assessing the sensibility of free-text responses. The research coordinator (KM) removed Wave 1, Time 1 responses received before January 12, 2024, identified as likely fraudulent if (1) multiple consecutive responses were submitted with identical start and end dates (to the min), (2) responses did not correspond to a unique IP address, (3) less than half (<50%) the questionnaire was completed, (4) responses did not correspond to a unique email address, (5) consecutive responses were submitted with a pattern of email addresses (eg, a repeating pattern of similar email addresses, such as “firstname.lastname123@samedomain.com”; non-English characters; random strings of letters and numbers), (6) responses were completed within an unrealistically short completion time (Time 1: <20 min and Time 2: <4 min), or (7) responses did not have a unique geolocation stamp (exported from Qualtrics) [1]. We recognized that these criteria could potentially screen out some valid participants; however, the large volume of likely fraudulent responses necessitated an approach that maximized the detection of fraudulent responses.

We emailed the Wave 1, Time 2 link to email addresses for participants who passed the Human Review for Wave 1, Time 1 (original Time 2 link). In the meantime, we developed new bot prevention and detection procedures, new survey links, and a new recruitment strategy, submitted as an amendment to the REB at the University of Toronto for a wave of recruitment on January 30, 2024 (Wave 2). Our amendment was approved on February 15, 2024 (Figure 1).

We continued to examine the integrity of the data collected during Wave 1, Time 1. We iteratively revised our screening criteria, balancing our need to maximize detection of fraudulent responses with our aim to minimize the risk of removing valid participant data. For example, we found that our original cutoff of ≤50% survey completion was overly sensitive (as determined by true participants reaching out when they did not receive the Time 2 link); we relaxed the threshold to ≤80% completion for Time 1 and ≤60% completion for Time 2. As we revised the criteria, we converted our Human Review into rules that were coded using R (Code Review; Table 1) [52]. We applied our Code Review to the responses from Wave 1 (Time 1) and compared it with our Human Review. This comparison identified 35 participants who were originally screened out by the Human Review but were later identified as likely legitimate respondents by Code Review. We sent a new Wave 1 (Time 2) link to those 35 participants from Wave 1 (Time 1) on March 29, 2024 (Figure 1).

**Table 1.** Rules for identifying fraudulent responses across 2 time points in the web-based survey (Code Review).

Wave and type of rule	Rules for identifying fraudulent responses in the Time 1 questionnaire	Rules for identifying fraudulent responses in the Time 2 questionnaire (administered 1 week later)
Wave 1		
Survey completeness	We removed responses: Rule 1.1: ≤80% complete.	We removed responses: Rule 2.1: ≤60% complete.
Inclusion criteria^a^	We removed responses: Rule 1.2: for which the respondent’s self-reported age was <18 years. Rule 1.3: submitted from outside of Canada, Ireland, the United Kingdom, or the United States.^b^	Not applicable.
Indicator of potential fraud	We removed responses: Rule 1.4: with a survey completion time (in s) identical to that of ≥3 other responses as recorded by Qualtrics (Qualtrics, LLC).^c^ Rule 1.5: that did not have a unique combination of survey start and end time stamps (to the min). Rule 1.6: for which the respondent’s self-reported date did not align with the survey start date recorded by Qualtrics (allowing for a 1-d grace period for time zone differences and human error). Rule 1.7: <20 minutes in duration.^d^ Rule 1.8: with duplicate email addresses across responses. Email address rules—unique to Wave 1 (Time 1). We removed responses: Rule 1.9: with strings of consecutive responses that shared the same email address format (eg, a pattern of letters plus digits) and identical email domains. Rule 1.10: if the participant’s email address contained a string of ≥5 digits. Rule 1.11: if the respondent’s email address contained a mix of upper- and lower-case letters (excluding if only the first letter is capitalized). Rule 1.12: if the respondent‘s email address contained abnormal or special characters (eg, #, ’, %, $, &, *, ?). Rule 1.13: if the respondent’s name contained abnormal or special characters (eg, #, �, %, $, &, *, ?).	We removed responses: Rule 2.2: with a survey completion time (in s) identical to that of ≥3 other responses as recorded by Qualtrics.^c^ Rule 2.3: that did not have a unique combination of survey start and end time stamps. Rule 2.4: for which the respondent’s self-reported date did not align with the survey start date recorded by Qualtrics (allowing for a 1-day grace period for time zone differences and human error). Rule 2.5: <4 minutes in duration.^d^ Rule 2.6: With duplicate email addresses across responses. Rule 2.7: if the email address did not correspond to an email address that had passed our checkpoints at Time 1 and been sent a link to Time 2 by the research coordinator.
Wave 2		
Survey completeness	We removed responses: Rule 1.1: <80% complete.	We removed responses: Rule 2.1: <60% complete.
Inclusion criteria^a^	We removed responses: Rule 1.2: for which the respondent’s self-reported age was <18 years old, per our inclusion criteria. Rule 1.3: submitted from outside of Canada, Ireland, the United Kingdom, or the United States, per our inclusion criteria.^b^	Not applicable.
Indicator of potential fraud	We removed responses: Rule 1.4: with a survey completion time (in s) identical to that of ≥3 other responses as recorded by Qualtrics.^c^ Rule 1.5: that did not have a unique combination of survey start and end time stamps (to the min). Rule 1.6: for which the respondent’s self-reported date did not align with the survey start date recorded by Qualtrics (allowing for a 1-d grace period for time zone differences and human error). Rule 1.7: <20 minutes in duration.^d^ Rule 1.8: with duplicate email addresses across responses. Unique to Wave 2. We removed responses: Rule 1.14: that contained answers to any of the honeypot questions. Rule 1.15: for which the email address corresponded to an email address that completed the Wave 1 questionnaires.	We removed responses: Rule 2.2: with a survey completion time (in s) identical to that of ≥3 other responses as recorded by Qualtrics.^c^ Rule 2.3: that did not have a unique combination of survey start and end time stamps. Rule 2.4: for which the respondent’s self-reported date did not align with the survey start date recorded by Qualtrics (allowing for a 1-d grace period for time zone differences and human error). Rule 2.5: <4 min in duration.^d^ Rule 2.6: with duplicate email addresses across responses. Rule 2.7: if the email address did not correspond to an email address that had passed our checkpoints at Time 1 and been sent a link to Time 2 by the research coordinator. Unique to Wave 2. We removed responses: Rule 2.8: that contained answers to any of the honeypot questions. Rule 2.9: for which the email address corresponded to an email address that completed the Wave 1 questionnaires.

a Screening for inclusion criteria (not necessarily for fraud).

b We were only able to apply this rule to responses that were 100% complete. Complete responses were tagged with latitude and longitude coordinates by Qualtrics, enabling us to identify the respondent’s geolocation.

c This rule is based on the assumption that participants had an unlimited amount of time to complete the survey questionnaire (given the questionnaire’s length and the flexibility to pause or take breaks). Because of this, the probability that 2 people would complete the survey in the same number of seconds is extremely low (nearly 0). Despite the low probability, 2 people can take the same amount of time due to coincidence. Therefore, we applied this rule with some flexibility (eg, it is acceptable for 2 people to have the same completion time).

d Time cutoffs were established based on feedback from members of the research team who piloted the questionnaires. During piloting, it took between 30 and 40 minutes to complete the Time 1 questionnaire and between 10 and 15 minutes to complete the Time 2 questionnaire.

#### Relaunch of Recruitment With Additional Bot Prevention and Mitigation Strategies: Wave 2

We launched Wave 2 with new links to Times 1 and 2 questionnaires on February 19, 2024. We applied revised and new recruitment, data collection, and bot prevention and detection strategies as approved by the REB at the University of Toronto (Figure 1). Refer to Table 1 for an overview of strategies to prevent and identify fraudulent responses in Waves 1 and 2.

### Strategies for Preventing Fraudulent Responses

#### Modifications to Recruitment Procedures

We made the following changes to our recruitment strategy: (1) we created new, uncompromised Qualtrics links [1]; (2) we stopped posting the links on public social media groups (community leads posted the links only in private social media groups for which new membership requests were monitored and validated by the community leads); (3) we removed mention of the token of appreciation from the recruitment poster and study materials; and (4) we administered gift cards at the end of the study, providing additional time for us to ensure responses met our final validity and quality checks for Time 1 and Time 2.

#### Modifications to Data Collection Tools

We added 4 honeypot questions to both the Time 1 and Time 2 questionnaires for Wave 2. Honeypot questions are HTML-coded questions that are not visible to humans, but are detectable by bots (and, depending on implementation, potentially by screen readers) [22]. Responses to honeypot questions are indicative of bots [22]. In addition, we added a question to the Wave 2 (Time 2) questionnaire asking about the participants’ country of residence. This question was added so that it could be cross-referenced with the response to the same question at Time 1 (inconsistent responses between Time 1 and Time 2 may indicate a fraudulent response). We applied the same Qualtrics survey protection settings as we did for Wave 1 [1].

#### Modifications to Bot Detection Strategies

We modified our Code Review for Wave 1 to use the new bot prevention features added to the Wave 2 questionnaires (honeypots, repeat question between Time 1 and Time 2) (Table 1). For Wave 2, we modified the code to be less strict than for Wave 1 because we were more confident in the integrity of our data after having improved our recruitment strategy for Wave 2. The new fraud-detection code for Wave 2 was applied across both time points.

#### Strategies for Identifying Fraudulent Responses

Our strategies for identifying fraudulent responses involved applying the final version of our Code Review to screen out responses that did not meet inclusion criteria (aged <18 years, lived in a country other than Canada, Ireland, the United Kingdom, or the United States), responses that were ≤80% complete for Time 1 and ≤60% complete for Time 2, and likely fraudulent responses as identified by survey completion time stamps, alignment of self-reported and survey-recorded dates, and characteristics of email addresses (Table 1). We tested and refined the code to remove all potentially fraudulent responses. Details of the iterations of the Code Review are described in Multimedia Appendix 3. The final version of the Code Review is described in Table 1.

Some of the criteria in the Code Review were primarily aimed at removing incomplete responses or responses that did not meet our inclusion criteria (age and country of residence) and may or may not indicate fraudulent responses (Table 1). However, we applied all of our screening criteria together, and the criteria are not mutually exclusive.

For Wave 1, we applied 13 rules for Time 1 and 7 rules for Time 2 (Table 1). Five of the rules were unique for Wave 1 (Time 1) and were designed to identify suspicious email addresses and names because screening email addresses has been recommended as one of the most effective strategies for identifying bots [6,22]. For Wave 2, we applied 10 rules for Time 1 and 7 rules for Time 2 (Table 1). We removed the rules for screening email addresses because we were more confident in the integrity of our data after having improved our recruitment strategy for Wave 2 and wanted to decrease our risk of excluding valid participants. Two of the rules in Wave 2, Times 1 and 2, were unique and were designed to detect responses to honeypot questions and to prevent repeat responses (intentional or accidental) from participants in Wave 1 (Table 1).

We recognized that it was possible for true participants to fail the Code Review. For example, due to memory issues commonly associated with Long COVID or human error, some participants may have unintentionally taken part in the study more than once. Since our aim was to remove all potentially fraudulent responses, we chose to accept this possibility. If participants contacted us via email to indicate they were awaiting the Time 2 link or expecting further results from the research, we cross-referenced their email addresses with the list of responses that we had screened out using the Code Review. If the participant seemed to be legitimate through correspondence and only failed minor aspects of the Code Review (eg, submitted 2 responses under the same email address), we would manually add that participant’s response back into the dataset.

### Ethical Considerations

The Long COVID and Episodic Disability Study was approved by the Health Sciences REB at the University of Toronto (protocol 41749) and the Saint James Hospital/Tallaght University Hospital Joint Research Ethics Committee (2024-Mar-34453445). The cover page of the Time 1 questionnaire included information about the purpose and rationale of the study, inclusion criteria, potential medical and social risks of participation, the voluntary nature of participation, the types of questions asked (questionnaires), and participants’ right to withdraw at any time. Both Time 1 and Time 2 questionnaires included an electronic eligibility and consent form. Eligible participants provided informed consent by clicking “I agree to participate in this research study.” Data were deidentified. Participants whose responses met the final validity and quality checks received a CAD $40 (CAD $1=US $0.72 as of June 15, 2026) or equivalent electronic gift card at the end of the study.

## Results

### Overview

**Figure 2.**
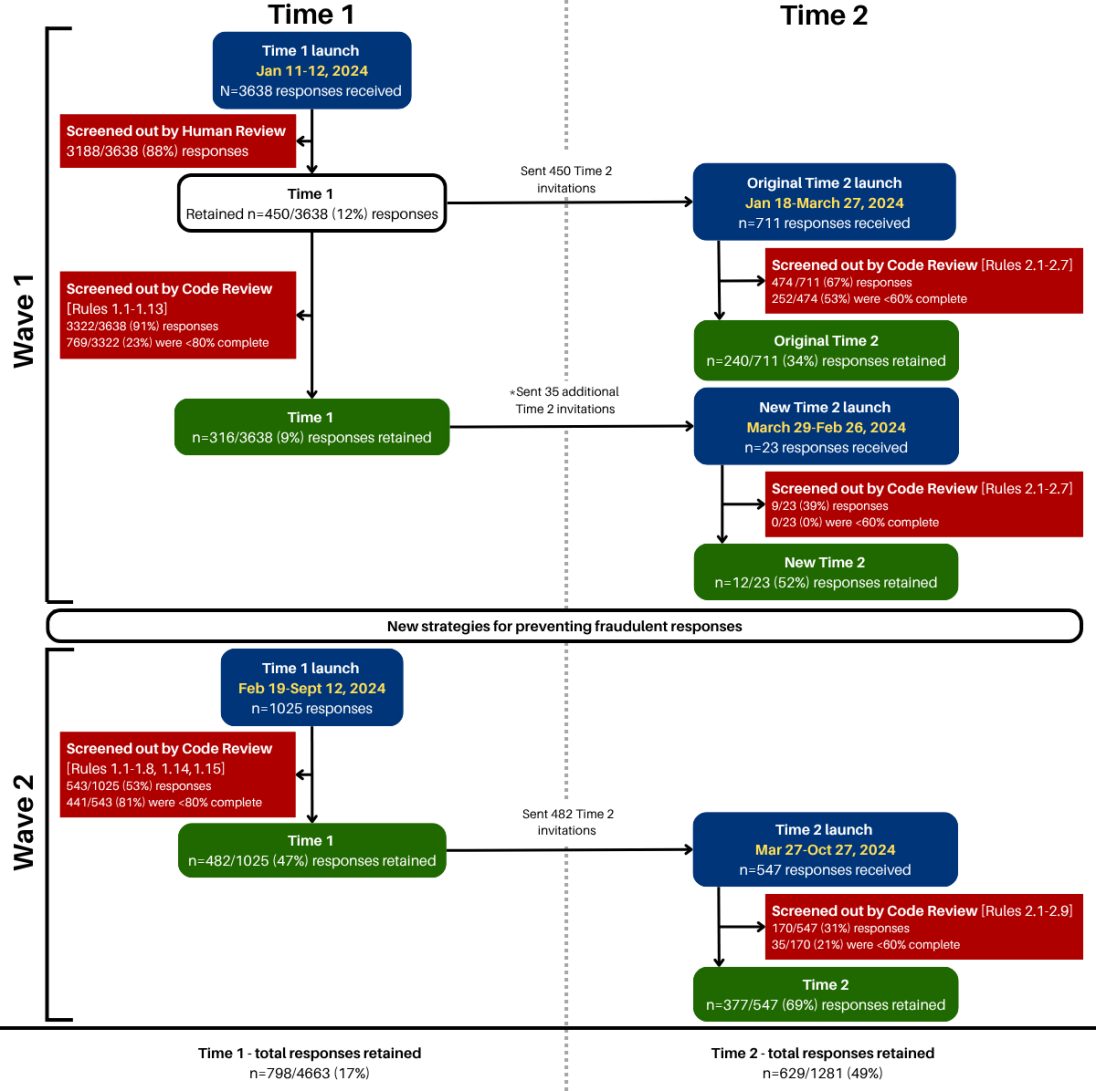
Flowchart of Waves 1 and 2 web-based survey responses.

We describe the resulting number of responses (defined as having viewed the survey, consented to participate, and completed at least 1 item on the questionnaire) across Waves 1 and 2 and Times 1 and Time 2 questionnaire administrations (Figure 2).

### Final Responses Retained

Across both waves, we retained 798 of 4663 (17%) responses for Time 1 and 629 of 1281 (49%) responses for Time 2 using our Code Review (Figure 2). Refer to Table 2 for a detailed breakdown of the number of responses removed based on each rule of the Code Review for Waves 1 and 2. These rules were applied together and were not mutually exclusive, and they may not always indicate fraudulent or bot-generated responses. For instance, many responses in Time 1 were removed because of questionnaire incompleteness, represented by ≤80% completion for Time 1 or ≤60% completion for Time 2.

**Table 2.** Results of responses removed by Code Review during Waves 1 and 2.

Rule no	Rule for identifying fraudulent responses	Wave 1 (failed rule and removed), n (%)	Wave 2 (failed rule and removed), n (%)
Time 1^a^			
1.1	≤80% complete.	769 (21)	441 (43)
1.2	For which the respondent’s self-reported age was <18 years, per our inclusion criteria.	646 (18)	44 (4)
1.3	Submitted from outside of Canada, Ireland, the United Kingdom, or the United States, per our inclusion criteria.^b^	309 (8)	33 (3)
1.4	With a survey completion time (min) identical to that of ≥3 other responses as recorded by Qualtrics (Qualtrics, LLC).	858 (24)	Not applicable
1.5	That did not have a unique combination of survey start and end time stamps.	Not applicable	0 (0)
1.6	For which the participant’s self-reported date did not align with the survey start date recorded by Qualtrics (allowing for a 1-d grace period for time zone differences and human error).	714 (20)	172 (17)
1.7	<20 minutes in duration.	1160 (32)	365 (36)
1.8	With duplicate email addresses.	62 (2)	23 (2)
1.9	Strings of consecutive responses that shared the same email address format (a pattern of letters plus digits) and identical email domains.	794 (22)	Not applicable
1.10	If the participant’s email address contained a string of ≥5 digits.	155 (4)	Not applicable
1.11	If the participant’s email address contained a mix of upper- and lower-case letters (excluding if only the first letter is capitalized).	317 (9)	Not applicable
1.12	If the participant’s email address contained abnormal or special characters (eg, #, ’, %, $, &, *, ?).	0 (0)	Not applicable
1.13	If the participant’s name contained abnormal or special characters (eg, #, �, %, $, &, *, ?).	338 (9)	Not applicable
1.14	Where the respondent had answered honeypot questions.	Not applicable	0 (0)
1.15	With email addresses that corresponded to respondents who had completed Wave 1.	Not applicable	202 (20)
Time 2^c^			
2.1	≤60% complete.	57 (8)	35 (6)
2.2	With a survey completion time (min) identical to that of ≥3 other responses as recorded by Qualtrics.	104 (14)	50 (9)
2.3	That did not have a unique combination of survey start and end time stamps.	Not applicable	0 (0)
2.4	For which the participant’s self-reported date did not align with the survey start date recorded by Qualtrics (allowing for a 1-d grace period for time zone differences and human error).	55 (8)	24 (4)
2.5	<4 minutes in duration.	54 (7)	28 (5)
2.6	With duplicate email addresses.	2 (<1)	30 (5)
2.7	If the email address did not correspond to an email address that had passed our checkpoints at Time 1 and had been sent a link to Time 2 by the research coordinator.	451 (61)	Not applicable
2.8	If the self-reported country did not match the self-reported country from Time 1.	Not applicable	97 (18)
2.9	That contained answers to any of the honeypot questions.	Not applicable	0 (0)

a Wave 1 (n=3638 responses) and Wave 2 (n=1025 responses).

b This rule could only be applied to responses that were 100% complete. Complete responses were tagged with latitude and longitude coordinates by Qualtrics, enabling identification of respondent geolocation.

c Wave 1 (n=734 responses) and Wave 2 (n=547 responses).

## Discussion

### Principal Findings

Our experiences conducting an international community-engaged web-based survey with adults living with Long COVID included substantial disruption caused by fraudulent responses and bots. Strategies implemented to prevent and mitigate the threat of fraudulent responses and bots included immediately closing our survey link and making swift amendments to our study protocol (including limiting our recruitment strategy and incorporating additional validity-check questions in our survey questionnaires). Concurrently, we established a set of rules to identify potentially fraudulent responses. Strategies for identifying fraudulent responses included monitoring response completion times, start and end time stamps, geolocation, and screening for suspicious email address characteristics and duplicates. We retained 17% of responses for Time 1 and 49% of responses for Time 2. While not all responses were removed because of fraudulent activity (many were removed because of incompleteness), we identified and removed a substantive number of responses attributed to suspected fraudulent activity. Furthermore, the 2 time points, 1 week apart, in our study design posed challenges as well as opportunities for preventing and detecting fraudulent responses.

Other research teams are experiencing similar challenges with fraudulent responses and bots when administering web-based surveys [9,13,18,22,24,27,29,53,54]. Although information about bots and web-based surveys is emerging for researchers to draw upon when designing web-based surveys, it is challenging for researchers to remain current given the rapidly evolving technology available to fraudulent actors, particularly in the context of generative AI [28-30,33,53,55-57]. This poses a risk to web-based survey research, as the rapid evolution and sophistication of the technology mean that research may continue to be vulnerable.

We outline lessons learned and recommendations for mitigating fraudulent- and bot-related disruptions and enhancing the integrity of web-based surveys. Our lessons learned can be categorized into (1) survey-design and implementation to reduce and identify fraudulent and bot-generated responses, (2) recruitment strategies to decrease the risk of web-based surveys being disrupted by fraudulent actors and bots, and (3) responding to disruptions caused by fraudulent actors and bots. We provide 11 recommendations (Table 3). We suggest the recommended strategies be used in combination to optimize the ability of research teams to prevent and identify bot-generated responses to web-based questionnaires.

**Table 3.** Lessons learned and recommendations for researchers to prevent and identify fraudulent, including bot-generated responses to online web-based questionnaires.

Lessons learned and recommendations for researchers	Type of strategy (prevention or mitigation)
Survey design and implementation to reduce and identify bot-generated responses	
Perform a review of the current literature, connect with other researchers performing web-based surveys, and liaise with the relevant Research Ethics Board about the newest threats and effective strategies before you design and launch an online survey.	Prevention
Inquire about and evaluate the information security technology used by survey software when selecting the platform to administer your online survey (eg, REDCap [Vanderbilt University Medical Center], Qualtrics [Qualtrics, LLC], and SurveyMonkey [SurveyMonkey Inc]). Invest in survey software with rigorous information security technology.	Prevention
Use bot-detection features embedded in survey software prior to launching web-based questionnaires. For example: (1) CAPTCHA^a^, (2) prevention of multiple responses from the same IP address, and (3) software-specific bot-detection (information security) features.	Prevention
Design questionnaire items to identify bots and fraudulent actors. For example: (1) honeypots, (2) repeated questions, and (3) open-ended questions.	Mitigation
Tailor screening criteria to the characteristics of the target population (eg, cognitive differences, intellectual disabilities, age-related factors, and language barriers).	Mitigation
Recruitment strategies to decrease the risk of web-based surveys being disrupted by bots	
Avoid recruitment in public social media groups.	Prevention
Engage community leads in tailored and targeted recruitment.	Prevention
Avoid advertising incentives for completing web-based questionnaires.	Prevention
Shut down corrupted links as quickly as possible.	Mitigation
Communicate with the Research Ethics Board about the disruption and strategies to mitigate the impacts of the disruption.	Mitigation
Use a combination of both manual (Human Review) and automated methods to screen for potentially fraudulent responses.	Mitigation

a CAPTCHA: Completely Automated Public Turing test to tell Computers and Humans Apart.

### Survey Design and Implementation to Reduce and Identify Bot-Generated Responses

Researchers should consider and explore information security features embedded in survey software that can assist in identifying bots and fraudulent actors before launching web-based questionnaires. For example, a CAPTCHA (Completely Automated Public Turing test to tell Computers and Humans Apart) may be added to a web-based questionnaire to assess whether a computer user is a human and rule out less sophisticated bots [22,58]. Unfortunately, this is not a foolproof method, as there is ever-evolving technology to decrypt reCAPTCHA [27,58]. Our questionnaire was corrupted by bots despite the use of a CAPTCHA at initial launch. While survey software also may include settings to prevent multiple responses from the same IP address, IP addresses may be obscured intentionally or unintentionally through the use of virtual private networks [22,27,58]. We were able to use IP addresses and associated data on geolocation to identify potentially fraudulent responses in conjunction with other strategies. Survey software companies, such as Qualtrics [1], also may have a bot-detection feature that analyzes responses and provides a score indicating how likely a response is to have been generated by a human. However, these bot-detection features are often moderately effective, can be costly, and should be used in combination with other strategies to protect the integrity of web-based questionnaires [22,27]. We applied these available features during our Wave 2 relaunch, which may have contributed to the improved integrity of our data.

Researchers may design questionnaire items to identify potential bots and fraudulent actors. For example, honeypots are questions that are programmed to engage bot respondents while being invisible to human respondents [17,22]. We added honeypots to our questionnaires in Wave 2; however, we did not receive any responses to these questions despite having other indicators of bots. Other research teams have had similar experiences, suggesting that most bots are now able to evade honeypots [22,59]. Including open-ended questions in web-based questionnaires provides opportunities to assess for potentially fraudulent responses, as researchers can check for identical or illogical responses to questions [23]. In our study, a research coordinator used this method in our early Human Review efforts for screening. It was time-consuming to apply to such a large volume of responses, and at times, difficult to systematically define what constituted an illogical response. As generative AI improves, it may become even more difficult to discern between human- and bot-generated text responses [15]. Given the vulnerabilities of each individual mitigation strategy, we recommend that these strategies be applied in combination. It is critical for researchers conducting web-based surveys to invest in survey platforms with rigorous and current cybersecurity measures to prevent breaches in survey integrity in the first place, as bot-detection strategies are ever-evolving in response to the evolving technology used to implement cyberattacks.

### Recruitment Strategies to Decrease the Risk of Web-Based Surveys Being Disrupted by Bots

Our experiences with this study taught us the importance of avoiding broad recruitment on social media, avoiding advertising incentives for completing web-based surveys, and engaging community leads in recruitment to reduce the risk of survey corruption by bots. Social media–based recruitment has advantages, notably the potential to reach demographically and geographically diverse groups at a relatively low cost [10,16]. However, fraudulent scams have become common on social media platforms [5,13,17]. The large volume of fraudulent responses we received during Wave 1 likely was influenced by our use of broad social media recruitment and mention of compensation in recruitment materials [5,17,22,28]. The number of fraudulent responses we received substantially decreased in Wave 2 when we only used private groups on social media (monitored by community leads) for recruitment and removed any mention of compensation from recruitment materials. Although avoiding advertising financial incentives may be a critical step to prevent bot-related disruptions to web-based surveys, it can also lead to less diversity among recruited participants [60]. Future research could explore whether other strategies regarding incentives, such as (but not limited to) lottery-based incentives, are effective at minimizing fraudulent responses while maintaining diversity among recruited participants. However, this may introduce issues related to compensating participants unequally. Engaging community leads in our recruitment efforts also helped us target our recruitment to real adults living with Long COVID, as community leads had established relationships within this population and were able to vet potential respondents.

### Responding to Disruptions Caused by Bots

Despite our preventative efforts to reduce fraudulent responses, we experienced an unprecedented influx of responses to our Time 1 questionnaire. Bots will respond to surveys quickly and repeatedly once they have access to a link [22]. When compromised, it is important to close links to web-based questionnaires as quickly as possible once they have been disrupted by bots [11,22]. Despite our relatively quick response (<24 h), we were left with thousands of responses to sort through, muddying the picture of which responses were valid.

We learned several strategies (both manual and automated) for screening data to identify bots and other potentially fraudulent responses. We adopted manual (Human Review) strategies, such as reviewing responses for suspicious names and email addresses, dates of survey completion, and duration of survey completion, that were similar to the approaches implemented by other research teams (Table 3) [13,22]. Manually screening for suspicious patterns in email addresses has been reported by other teams as one of the most effective [13,22], though time- and resource-intensive [24], strategies for identifying bots. A unique aspect of our approach was that we developed code in R (Code Review) to replicate our Human Review [52], while adding new automatic screening criteria. This allowed us to apply our screening criteria efficiently and in a standardized way across all responses we received. A combination of both manual and automatic screening is essential for identifying and removing bot-generated data [13,18,22,24,34].

When responding to disruptions caused by fraudulent and bot-generated responses, it is important to consider both the sensitivity (correctly detecting fraudulent responses) and specificity (correctly identifying true human respondents) of the methodology. It is difficult to achieve high levels of both sensitivity and specificity at the same time, as methods that improve one may reduce the performance of the other. Researchers should navigate the balance between the two, using strategies such as question design, checking survey completion times, and analyzing patterns in responses to classify fraudulent and true responses. In this study, we prioritized sensitivity. This increased our risk of removing true participant responses from the dataset. However, this was necessary to maximize the integrity of our dataset. Regular meetings with the community leads were invaluable for guiding our approach.

### Strengths and Limitations

Given that fraudulent- and bot-related disruptions to web-based surveys are common among research teams, it is important to document and communicate strategies to prevent and mitigate fraudulent responses [9,13,18,22,24,29]. Strengths of our study included our documented methodical approach to identifying and handling bots and other potentially fraudulent responses. We systematically identified fraudulent responses using a rigorously developed set of automated rules (Code Review). Future research teams using web-based surveys may apply similar strategies. Another strength was our community-engaged approach to participant recruitment. The 5 community groups involved in this study were instrumental in troubleshooting our recruitment strategy, helping us shift to more targeted recruitment methods such as word of mouth, listservs, and private social media groups while still being able to reach our target population of adults living with Long COVID [61].

This web-based survey involved 2 time points of questionnaire administration, 1 week apart. Therefore, when the Wave 1 (Time 1) questionnaire was disrupted by bots, we had to act fast to determine who to invite to complete the Wave 1 (Time 2) questionnaire with the original link (and screen out potentially fraudulent responses). Since the Human Review was manual and time-consuming, we iteratively revised our screening process several times before we developed our Code Review. By that time, we had already sent invitations to some likely fraudulent actors, as indicated by a greater number of Time 2 responses than invitations sent in Wave 1, and screened out some likely legitimate participants (Figure 2). As a result, we created a new link to access the Wave 1 (Time 2) questionnaire to capture as many of our legitimate participants as possible. In other ways, having the second time point to our web-based survey was valuable, allowing us to cross-reference responses between Time 1 and Time 2 to check the quality and effectiveness of our screening approaches (eg, consistency of country of residence between Time 1 and Time 2).

Although our findings are specific to our experiences with a 2-time point survey among people living with Long COVID, the lessons learned and recommendations from this work may be applicable to researchers conducting international community-engaged web-based surveys across other conditions. Strategies to prevent and mitigate fraudulent and bot-generated responses should be tailored to the intended research population using community-engaged approaches throughout. For example, we decided to avoid the use of logic-check strategies given the high prevalence of cognitive challenges among people living with Long COVID [62,63]. For other populations, logic-check strategies may be useful and effective for identifying bots. The recommendations from this work build upon the recommendations of other teams and existing guidelines [18-20,22,23,26,28,29,34,51].

### Implications for Future Research

It is likely impossible to prevent all risk of disruption by fraudulent actors and bots when using a web-based survey research design. However, best efforts should be made to reduce this risk and protect the integrity of data collected through web-based questionnaires. It is essential for computer science researchers and coders to develop effective and affordable anti-bot or anti-AI technology that can help protect web-based questionnaires from these threats. Researchers should regularly evaluate their bot-prevention and screening strategies throughout the study design, data collection, and screening. Researchers should also transparently report their process for ensuring data integrity when publishing results from web-based surveys and remain in close correspondence with the relevant REB throughout. Peer reviewers and journal editors should also consider whether and how researchers using web-based surveys have reported their methods for mitigating fraudulent responses during the peer-review process. In addition to threatening data integrity, bot-related disruption may influence the experiences of legitimate research participants. For example, in our study, we were at times delayed in sending invitations to the Time 2 questionnaires, as outlined in our study protocol, and in administering the token of appreciation to participants. It is possible that these disruptions could cause stress among participants or decrease their trust in researchers or community leads involved in recruitment. Therefore, researchers should address risks associated with bot infiltration and bot-screening processes with participants as part of informed consent processes.

### Conclusions

Despite the benefits of web-based survey study designs, the risk of fraudulent responses threatens the integrity of online research. Prevention and detection of fraudulent responses may become increasingly difficult because of bot- and AI-assisted fraud. Researchers should proactively plan for the prevention and detection of fraudulent responses, including bots. Their strategies should be well-documented and reported so that researchers can learn from one another and adapt their strategies to keep pace with technological advances in bots and AI. The strategies we have documented and recommended here may be useful to other research communities, but are also specific to our study and experiences. We recommend that research teams implement multiple bot-prevention and detection strategies that are systematic and suited to each study design and study population.

## Supplementary material

10.2196/88838 Multimedia Appendix 1 Initial recruitment materials.

10.2196/88838 Multimedia Appendix 2 Invitation to Time 2 questionnaire.

10.2196/88838 Multimedia Appendix 3 Iterative Code Review strategies for identifying fraudulent responses.

## Abbreviations

AI: artificial intelligence
CAPTCHA: Completely Automated Public Turing test to tell Computers and Humans Apart
EDQ: Episodic Disability Questionnaire
REB: Research Ethics Board
