# Supplementary material for: Battling the Bots and Defending Against Fraudulent Responses in an International Community-Engaged Web-Based Survey With People Living With Long COVID: Methodological Study
Source: J Med Internet Res. 2026 Jul 23;28:e88838. doi: 10.2196/88838 (PMC13395426; doi:10.2196/88838)
Supplement: Multimedia Appendix 1 [file jmir-v28-e88838-s001.pdf]

Poster to be attached as PDF in email, newsletters

Long COVID and Episodic Disability – Research Study

Are you a person living with Long COVID?

We are conducting a study to **enhance understanding of the experiences of adults living with Long COVID**, and **to assess a new questionnaire for its ability to measure the types of disability experienced by people living with Long COVID**, such as fatigue, pain, and challenges with day-to-day activities. For the second part of the study, we would like to trial a questionnaire that measures episodic disability with adults living with Long COVID. If you participate, you will be asked to complete the **Episodic Disability Questionnaire (EDQ), the Long COVID EDQ Supplement (LC-EDQ Suppl)**, an **online demographic questionnaire**, and **five additional online general health status questionnaires**, which will take approximately **30-40 minutes** in total. Additionally, **one week later** we will ask you to complete **only the EDQ and LC-EDQ Suppl** again, which will take approximately **10-15 minutes** to complete.

If you are interested in participating in the study or obtaining more information, please contact the Research Coordinator for your country:

- **Canada or United States:** Kiera McDuff at kiera.mcduff@mail.utoronto.ca or 416-946-3935**.**
- **Ireland or United Kingdom:** Natalie Sullivan at n.stclair-sullivan@nhs.net

This study has been approved by the **University of Toronto Research Ethics Board** (Protocol #41749).

This study is funded by the **Canadian Institutes of Health Research (CIHR), Emerging COVID-19 Research Gaps and Priorities Funding Opportunity**
(FRN: GA4-177753).

Twitter Recruitment

Images for Twitter:

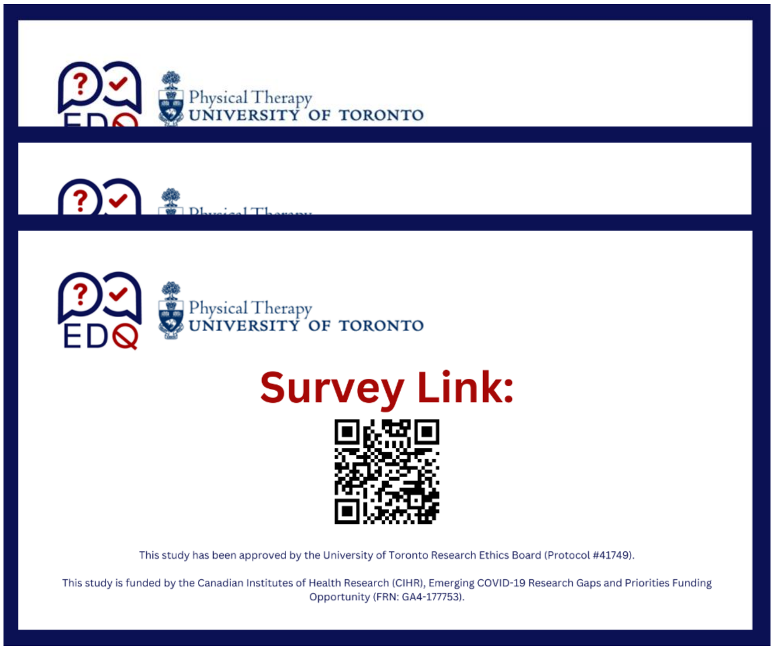

Text for Twitter:
Seeking adults living with #LongCOVID in Canada, Ireland, the US and the UK to participate in the Long COVID and Episodic Disability Study. The aim is to assess a questionnaire for its ability to measure disability experienced by people with Long COVID. Click the link to learn more and participate: [insert link]

Instagram Recruitment

Images for Instagram:

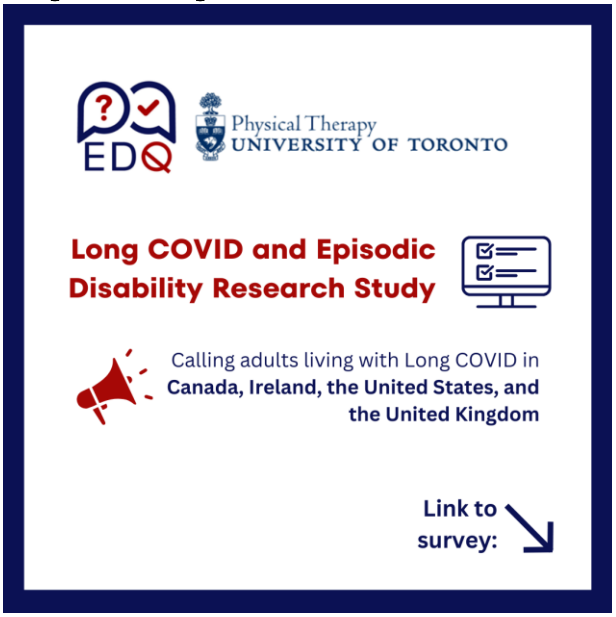

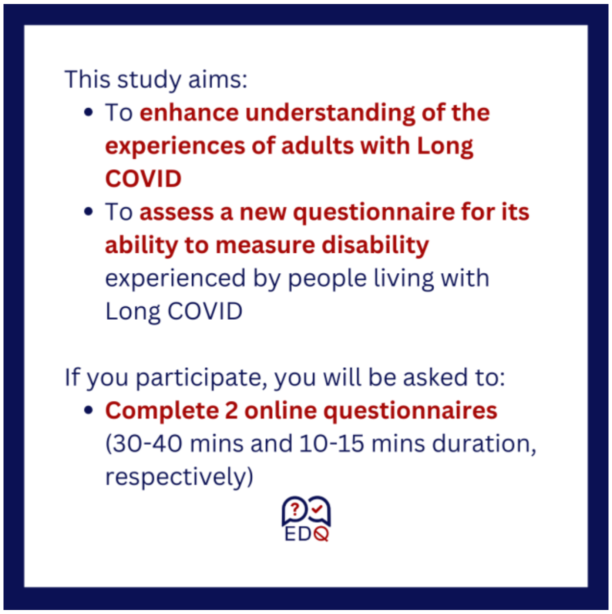

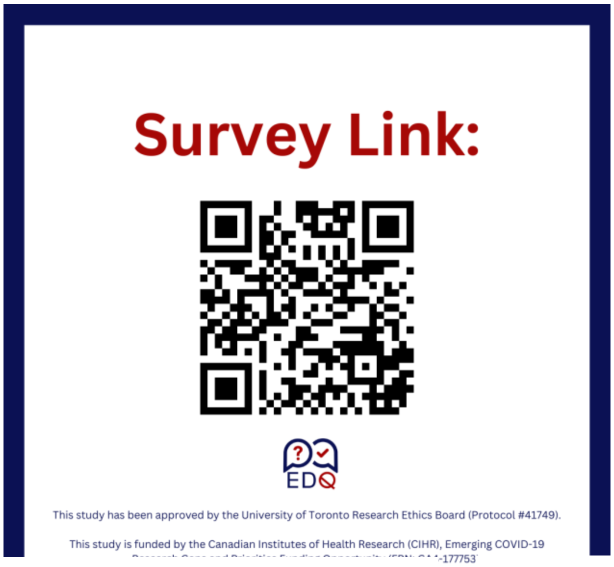

Text for Instagram:
Seeking adults living with [illegible] #LongCOVID in Canada,
Ireland, the US and the [illegible] UK to participate in the
Long COVID and Episodic [illegible] Disability Study. The aim
is to assess a [illegible] questionnaire for its
ability to measure [illegible] disability experienced by
people with Long COVID. [illegible] Click the link to learn
more and participate: [illegible] [insert link]

Initial recruitment email

Subject [illegible] Line – **Research**
Study [illegible] **Participation: Long**
COVID [illegible] **and Episodic**
Disability [illegible] **Study**

Hello (insert potential participant name here),

Thank you for your interest in the research study titled **"Long COVID and Episodic Disability: Advancing the Conceptualization, Measurement and Knowledge of Episodic Disability with people living with Long COVID."**

Our study aims to gain a better understanding of the disability experiences among people living with Long COVID and to assess how well the Episodic Disability Questionnaire (EDQ) (which captures health-related challenges experienced by adults living with episodic disability) and a newly developed Long COVID EDQ Supplement (LC-EDQ Suppl) measure the presence, severity and episodic nature of disability among people living with Long COVID. The LC-EDQ Suppl was developed during the first phase of our study, which involved interviews with adults living with Long COVID, and now we would like to test the questionnaire with adults living with Long COVID. To participate in this study, you will be asked to complete the **online EDQ**, the **LC-EDQ Suppl**, a **demographic questionnaire**, and **five additional online questionnaires**, which will take approximately 30-45 minutes in total. Additionally, **one week later** we will ask you to complete **only the EDQ and LC-EDQ Suppl** again, which will take approximately 10-15 minutes to complete.

After completion of the second EDQ and LC-EDQ Suppl, you will receive $40 CAD / £25 / $30 USD / €25 e-gift card as a token of appreciation for your participation in this study.

Here is the link to the study information and consent page and the initial set of questionnaires:

(insert T1 Qualtrics link here).

If you have any questions about the research study, please contact:

> **For Canada or United States:** Kiera McDuff (Research Coordinator) at kiera.mcduff@mail.utoronto.ca or 416-946-3935.
>
> **For Ireland or United Kingdom:** Natalie St. Clair-Sullivan at n.stclair-sullivan@nhs.net

Thank you for your interest in participating in this study.

Regards,

Kiera McDuff
BSc Kin, MScPT
Research Coordinator
Department of Physical Therapy
University of Toronto
500 University Avenue
Toronto, ON, M5G 1V7 Canada
Email: kiera.mcduff@mail.utoronto.ca
Phone: 416-946-3935

This study is funded by the **Canadian Institutes of Health Research (CIHR), Emerging COVID-19 Research Gaps and Priorities Funding Opportunity (FRN: GA4-177753).**

CIHR IRSC
