# Supplementary material for: Battling the Bots and Defending Against Fraudulent Responses in an International Community-Engaged Web-Based Survey With People Living With Long COVID: Methodological Study
Source: J Med Internet Res. 2026 Jul 23;28:e88838. doi: 10.2196/88838 (PMC13395426; doi:10.2196/88838)
Supplement: Multimedia Appendix 2 [file jmir-v28-e88838-s002.pdf]

Subject Line – Research Study Participation: Long COVID and Episodic Disability Study

Hello (insert potential participant name here),

Thank you for completing the first step of our research study titled **“Long COVID and Episodic Disability: Advancing the Conceptualization, Measurement and Knowledge of Episodic Disability with people living with Long COVID.”**

We are now following up one week after you completed the first set of questionnaires to provide you with the information you need to complete the final Episodic Disability Questionnaire (EDQ) and Long COVID EDQ Supplement (LC-EDQ Suppl) as the final step for the study. We estimate that completing **only the EDQ and LC-EDQ Suppl** in this final step will take approximately 10-15 minutes.

After you complete this second EDQ and LC-EDQ Suppl, we will follow up with you regarding your $40 CAD / £25 / $30 USD / €25 e-gift card as a token of appreciation for your participation in this study.

Here is the link to the study information and consent page for the second step and to the second EDQ and LC-EDQ Suppl:

(insert T2 Qualtrics link here).

If you have any questions, feel free to contact myself, the research coordinator, at kiera.mcduff@mail.utoronto.ca or (416) 946-3935.

Thank you for your interest in participating in this study.

Regards,

Kiera McDuff
BSc Kin, MScPT
Research Coordinator
Department of Physical Therapy
University of Toronto
500 University Avenue
Toronto, ON, M5G 1V7 Canada
Email: kiera.mcduff@mail.utoronto.ca
Phone: 416-946-3935

This study is funded by the **Canadian Institutes of Health Research (CIHR), Emerging COVID-19 Research Gaps and Priorities Funding Opportunity (FRN: GA4-177753).**

Date Last Revised: December 11, 2025
