# Supplementary material for: Battling the Bots and Defending Against Fraudulent Responses in an International Community-Engaged Web-Based Survey With People Living With Long COVID: Methodological Study
Source: J Med Internet Res. 2026 Jul 23;28:e88838. doi: 10.2196/88838 (PMC13395426; doi:10.2196/88838)
Supplement: Multimedia Appendix 3 [file jmir-v28-e88838-s003.pdf]

Code for Identifying Fraudulent Responses –Version One

We applied the first version of the code to the initial wave (Wave One) of responses we received to the Time One questionnaire on January 22nd, 2024 and to Wave One of the responses we received to the Time Two questionnaire on January 29th, 2024. See Table 1 for the rules for version one of the code.

Table 1: *Code for Identifying Fraudulent Responses – Wave One, Version One*

Code for Identifying Fraudulent Reponses in Time One Questionnaire	Code for Identifying Fraudulent Reponses in Time Two Questionnaire
1) We removed responses for which the respondent's geolocation did not align with their self-reported country of residence – Canada, Ireland, UK, or US*<br>2) We removed responses with duplicate email addresses.<br>3) We removed responses for which the participant's self-reported date did not align with the survey start date recorded by Qualtrics (allowing for a 1-day grace period for time zone differences and human error).<br>4) We removed responses for which the respondent's self-reported age was <18 years old, per our inclusion criteria.<br>5) We removed responses for which the participant's self-reported year of COVID-19 infection that led to Long COVID was prior to January 2020.<br>6) We removed responses for which the participant's self-reported duration of living with Long COVID did not align with their self-reported year of COVID-19 infection.<br>7) We removed responses with duplicate survey completion durations (in seconds).<br>8) We removed responses that did not have a unique combination of survey start and end time stamps.<br>9) We removed responses that were <20 minutes in duration.<br>10) We removed responses were <80% complete.	1) We removed responses if the email address did not correspond to an email address that had passed our check points at Time One and been sent a link to Time Two by the research coordinator.<br>2) We removed responses with duplicate email addresses.<br>3) We removed responses for which the participant's self-reported date did not align with the survey start date recorded by Qualtrics (allowing for a 1-day grace period for time zone differences and human error).<br>4) We removed responses for which the geolocation recorded at Time Two did not align with the self-reported country from Time One.<br>5) We removed responses with duplicate survey completion durations (in seconds).<br>6) We removed responses that did not have a unique combination of survey start and end time stamps.<br>7) We removed responses that were <4 minutes in duration.<br>8) We removed responses <60% complete.

*Only able to apply to responses that were 100% complete as geolocation data was not recorded by Qualtrics for incomplete responses

Code for Identifying Fraudulent Responses – Version Two

As we continued to recruit additional participants and evaluate our data, we determined that some of the criteria in our code may be too strict and failing to account for human error in responding to the questionnaire, resulting in removal of likely valid responses to the questionnaires. We also began to receive a number of emails from people who were recruited in Wave One, stating that they had completed the questionnaire but not yet received a gift card. On March 13th, 2024, we revised our code for Wave One and developed code for Wave Two (see Table 2).

Table 2: *Code for Identifying Fraudulent Responses – Version Two*

Wave	Code for Identifying Fraudulent Reponses in Time One Questionnaire	Code for Identifying Fraudulent Reponses in Time Two Questionnaire
Wave One	1) We removed responses for which the respondent's geolocation did not align with their self-reported country of residence – Canada, Ireland, UK, or US*<br>2) We removed responses with duplicate email addresses.<br>3) We removed responses for which the participant's self-reported date did not align with the survey start date recorded by Qualtrics (allowing for a 1-day grace period for time zone differences and human error).<br>4) We removed responses for which the respondent's self-reported age was <18 years old, per our inclusion criteria.<br>~~5) We removed responses for which the participant's self-reported year of COVID-19 infection that led to Long COVID was prior to January 2020.~~<br>~~6) We removed responses for which the participant's self-reported duration of living with Long COVID did not align with their self-reported year of COVID-19 infection.~~<br>~~7) We removed responses with duplicate survey completion durations (in seconds).~~<br>~~8) We removed responses that did not have a unique combination of survey start and end time stamps.~~<br>9) We removed responses that were <20 minutes in duration.	1) We removed responses if the email address did not correspond to an email address that had passed our check points at Time One and been sent a link to Time Two by the research coordinator.<br>2) We removed responses with duplicate email addresses.<br>3) We removed responses for which the participant's self-reported date did not align with the survey start date recorded by Qualtrics (allowing for a 1-day grace period for time zone differences and human error).<br>4) We removed responses for which the geolocation recorded at Time Two did not align with the self-reported country from Time One.<br>~~5) We removed responses with duplicate survey completion durations (in seconds).~~<br>~~6) We removed responses that did not have a unique combination of survey start and end time stamps.~~<br>7) We removed responses that were <4 minutes in duration.<br>8) We removed responses <60% complete.

Wave	Code for Identifying Fraudulent Reponses in Time One Questionnaire	Code for Identifying Fraudulent Reponses in Time Two Questionnaire
	10) We removed responses were <80% complete.	
Wave Two	1) We removed responses for which the respondent's geolocation did not align with their self-reported country of residence – Canada, Ireland, UK, or US*<br>2) We removed responses with duplicate email addresses.<br>3) We removed responses for which the participant's self-reported date did not align with the survey start date recorded by Qualtrics (allowing for a 1-day grace period for time zone differences and human error).<br>4) We removed responses for which the respondent's self-reported age was <18 years old, per our inclusion criteria.<br>5) We removed responses that were <20 minutes in duration.<br>6) We removed responses were <80% complete.<br>7) We removed any responses for which the email address corresponded to an email address that completed the Wave One questionnaires.	1) We removed responses if the email address did not correspond to an email address that had passed our check points at Time One and been sent a link to Time Two by the research coordinator.<br>2) We removed responses with duplicate email addresses.<br>3) We removed responses for which the participant's self-reported date did not align with the survey start date recorded by Qualtrics (allowing for a 1-day grace period for time zone differences and human error).<br>4) We removed responses for which the participant's self-reported country at Time Two did not align with the self-reported country from Time One.<br>~~5) We removed responses with duplicate survey completion durations (in seconds).~~<br>~~6) We removed responses that did not have a unique combination of survey start and end time stamps.~~<br>7) We removed responses that were <4 minutes in duration.<br>8) We removed responses <60% complete.<br>9) We removed any responses that contained answers to any of the honeypot questions.<br>10) We removed responses if the email address did not correspond to an email address that had passed our check points at Time One and been sent a link to Time Two by the research coordinator.

*Only able to apply to responses that were 100% complete as geolocation data was not recorded by Qualtrics for incomplete responses

Code for Identifying Fraudulent Responses – Version Three

On March 21, 2024, applying Version Two of our code, we cross-checked the results of our code with a human screen (focused on identifying suspicious email addresses) of responses. We noted that there were still a large number of responses with suspicious email addresses that were passing our code (Version Two). We examined those responses that were flagged as potentially suspicious by the human screen for patterns. We also developed code to mimic the human screen of email addresses. On March 28, 2024, we modified our code again, to be stricter and to apply the rules for screening email addresses. See Table 3.

Table 3: *Code for Identifying Fraudulent Responses – Version Three*

Wave	Code for Identifying Fraudulent Reponses in Time One Questionnaire	Code for Identifying Fraudulent Reponses in Time Two Questionnaire
Wave One	Email address rules:<br>1) We removed strings of responses that were submitted consecutively that shared the same email address format (a pattern of letters plus digits) and identical email domains.<br>2) We removed responses if the participant's email address contained a string of ≥5 digits.<br>3) We removed responses if the participant's email address contained a mix of upper- and lower-case letters (excluding if only the first letter is capitalized).<br>4) We removed responses if the participant's email address contained abnormal or special characters (e.g., #, ', %, $, &, *, ?).<br>5) We removed responses if the participant's name contained abnormal or special characters (e.g., #, �, %, $, &, *, ?).<br><br>Other rules:<br>1) We removed responses for which the respondent's geolocation did not align with their self-reported country	1) We removed responses if the email address did not correspond to an email address that had passed our check points at Time One and been sent a link to Time Two by the research coordinator.<br>2) We removed responses with duplicate email addresses.<br>3) We removed responses for which the participant's self-reported date did not align with the survey start date recorded by Qualtrics (allowing for a 1-day grace period for time zone differences and human error).<br>4) We removed responses for which the geolocation recorded at Time Two did not align with the self-reported country from Time One.<br>5) We removed responses that were <4 minutes in duration.<br>6) We removed responses <60% complete.<br>7) We removed responses with ≥2 duplicate survey completion time (in minutes) as recorded by Qualtrics.

Wave	Code for Identifying Fraudulent Reponses in Time One Questionnaire	Code for Identifying Fraudulent Reponses in Time Two Questionnaire
	of residence – Canada, Ireland, UK, or US*<br>2) We removed responses with duplicate email addresses.<br>3) We removed responses for which the participant's self-reported date did not align with the survey start date recorded by Qualtrics (allowing for a 1-day grace period for time zone differences and human error).<br>4) We removed responses for which the respondent's self-reported age was <18 years old, per our inclusion criteria.<br>5) We removed responses that were <20 minutes in duration.<br>6) We removed responses were <80% complete.<br>7) We removed responses with ≥2 duplicate survey completion time (in minutes) as recorded by Qualtrics.	
Wave Two	Email address rules:<br>8) We removed strings of responses that were submitted consecutively that shared the same email address format (a pattern of letters plus digits) and identical email domains.<br>9) We removed responses if the participant's email address contained a string of ≥5 digits.<br>10) We removed responses if the participant's email address contained a mix of upper- and lower-case letters (excluding if only the first letter is capitalized).<br>11) We removed responses if the participant's email address contained abnormal or special characters (e.g., #, ', %, $, &, *, ?).<br>12) We removed responses if the participant's name contained abnormal or special characters (e.g., #, �, %, $, &, *, ?).<br><br>Other rules:	1) We removed responses if the email address did not correspond to an email address that had passed our check points at Time One and been sent a link to Time Two by the research coordinator.<br>2) We removed responses with duplicate email addresses.<br>3) We removed responses for which the participant's self-reported date did not align with the survey start date recorded by Qualtrics (allowing for a 1-day grace period for time zone differences and human error).<br>4) We removed responses for which the participant's self-reported country at Time Two did not align with the self-reported country from Time One.<br>~~5) We removed responses with duplicate survey completion durations (in seconds).~~<br>~~6) We removed responses that did not have a unique combination of survey start and end time stamps.~~

Wave	Code for Identifying Fraudulent Reponses in Time One Questionnaire	Code for Identifying Fraudulent Reponses in Time Two Questionnaire
	1) We removed responses for which the respondent's geolocation did not align with their self-reported country of residence – Canada, Ireland, UK, or US*<br>2) We removed responses with duplicate email addresses.<br>3) We removed responses for which the participant's self-reported date did not align with the survey start date recorded by Qualtrics (allowing for a 1-day grace period for time zone differences and human error).<br>4) We removed responses for which the respondent's self-reported age was <18 years old, per our inclusion criteria.<br>5) We removed responses that were <20 minutes in duration.<br>6) We removed responses were <80% complete.<br>7) We removed any responses for which the email address corresponded to an email address that completed the Wave One questionnaires.<br>8) We removed responses with ≥2 duplicate survey completion time (in minutes) as recorded by Qualtrics.<br>9) The participant did not respond to any of the honeypot questions	7) We removed responses that were <4 minutes in duration.<br>8) We removed responses <60% complete.<br>9) We removed any responses that contained answers to any of the honeypot questions.<br>10) We removed responses if the email address did not correspond to an email address that had passed our check points at Time One and been sent a link to Time Two by the research coordinator.<br>11) We removed responses with ≥2 duplicate survey completion time (in minutes) as recorded by Qualtrics.<br>12) The participant did not respond to any of the honeypot questions

*Only able to apply to responses that were 100% complete as geolocation data was not recorded by Qualtrics for incomplete responses

Code for Identifying Fraudulent Responses – Version Four (Final Version)
During the process of data cleaning, we identified that our rule to remove "responses with ≥2 duplicate survey completion time (in minutes) as recorded by Qualtrics" was too strict. Participants who had initially been screened out due to that rule were reaching out to research team and requesting to be sent the Time Two link. Therefore, on March 29, 2024, we modified our code a final time, such that "We removed responses with ≥3 duplicate survey completion time (in minutes) as recorded by Qualtrics." See Table 4.

Table 4: Code for Identifying Fraudulent Responses – Version Four (Final Version)

Wave	Rules for Identifying Fraudulent Reponses in Time One Questionnaire	Rules for Identifying Fraudulent Reponses in Time Two Questionnaire
One	1) We removed responses were <80% complete.<br>2) We removed responses for which the respondent's self-reported age was <18 years old, per our inclusion criteria.*<br>3) We removed responses that were submitted from outside of Canada, Ireland, UK, or US, per our inclusion criteria. We were only able to apply this rule to responses that were 100% complete; complete responses were tagged with a latitude and longitude by Qualtrics which enabled us to identify the geolocation of the respondent.*<br>4) We removed responses with ≥3 duplicate survey completion time (in minutes) as recorded by Qualtrics.ǂ<br>5) We removed responses that did not have a unique combination of survey start and end time stamps.<br>6) We removed responses for which the participant's self-reported date did	1) We removed responses <60% complete.<br>2) We removed responses with ≥3 duplicate survey completion time (in minutes) as recorded by Qualtrics.ǂ<br>3) We removed responses that did not have a unique combination of survey start and end time stamps.<br>4) We removed responses for which the participant's self-reported date did not align with the survey start date recorded by Qualtrics (allowing for a 1-day grace period for time zone differences and human error).<br>5) We removed responses that were <4 minutes in duration.†<br>6) We removed responses with duplicate email addresses.<br>7) We removed responses if the email address did not correspond to an email address that had passed our check points at Time One and been

Wave	Rules for Identifying Fraudulent Reponses in Time One Questionnaire	Rules for Identifying Fraudulent Reponses in Time Two Questionnaire
	not align with the survey start date recorded by Qualtrics (allowing for a 1-day grace period for time zone differences and human error).<br>7) We removed responses that were <20 minutes in duration.[†]<br>8) We removed responses with duplicate email addresses.<br><br>Email address rules:<br>9) We removed strings of responses that were submitted consecutively that shared the same email address format (a pattern of letters plus digits) and identical email domains.<br>10) We removed responses if the participant's email address contained a string of ≥5 digits.<br>11) We removed responses if the participant's email address contained a mix of upper- and lower-case letters (excluding if only the first letter is capitalized).<br>12) We removed responses if the participant's email address contained abnormal or special characters (e.g., #, ', %, $, &, *, ?).<br>13) We removed responses if the participant's name contained abnormal or special characters (e.g., #, �, %, $, &, *, ?).	sent a link to Time Two by the research coordinator.
Two	1) We removed responses were <80% complete.<br>2) We removed responses for which the respondent's self-reported age was <18 years old, per our inclusion criteria.*<br>3) We removed responses that were submitted from outside of Canada, Ireland, UK, or US, per our inclusion criteria. We were only able to apply this rule to responses that were 100% complete; complete responses were tagged with a latitude and longitude by	1) We removed responses <60% complete.<br>2) We removed responses with ≥3 duplicate survey completion time (in minutes) as recorded by Qualtrics.[‡]<br>3) We removed responses that did not have a unique combination of survey start and end time stamps.<br>4) We removed responses for which the participant's self-reported date did not align with the survey start date recorded by Qualtrics (allowing for a 1-day grace period for time zone differences and human error).

Date last revised: December 3, 2025

Wave	Rules for Identifying Fraudulent Reponses in Time One Questionnaire	Rules for Identifying Fraudulent Reponses in Time Two Questionnaire
	Qualtrics which enabled us to identify the geolocation of the respondent.*<br>4) We removed responses with ≥3 duplicate survey completion time (in minutes) as recorded by Qualtrics.‡<br>5) We removed responses that did not have a unique combination of survey start and end time stamps.<br>6) We removed responses for which the participant's self-reported date did not align with the survey start date recorded by Qualtrics (allowing for a 1-day grace period for time zone differences and human error).<br>7) We removed responses that were <20 minutes in duration.†<br>8) We removed responses with duplicate email addresses.<br>9) We removed any responses that contained answers to any of the honeypot questions.<br>10) We removed any responses for which the email address corresponded to an email address that completed the Wave One questionnaires.	5) We removed responses that were <4 minutes in duration.†<br>6) We removed responses with duplicate email addresses.<br>7) We removed responses if the email address did not correspond to an email address that had passed our check points at Time One and been sent a link to Time Two by the research coordinator.<br>8) We removed any responses that contained answers to any of the honeypot questions.<br>9) We removed any responses for which the email address corresponded to an email address that completed the Wave One questionnaires.

*Screening for the inclusion criteria (not necessarily for fraud).
**We were only able to apply this rule to responses that were 100% complete; complete responses were tagged with a latitude and longitude by Qualtrics which enabled us to identify the geolocation of the respondent.
‡This rule is based on the assumption that participants had an unlimited amount of time to complete the survey questionnaire (given the questionnaire's length and the flexibility to pause or take breaks). Because of this, the probability that two people would complete the survey in exactly the same number of seconds is extremely low (nearly 0). Despite the low probability, it is possible for two people to take the same amount of time due to coincidence. Therefore, we applied this rule with some flexibility (for example, it is acceptable for two people to have the same completion time).
†Time cut offs were established based on feedback we received from members of the research team who had piloted the questionnaires; in the piloting process, it took between 30-40 minutes to complete the Time One questionnaire and between 10-15 minutes to complete the Time Two questionnaire.
